# Coverage of isoniazid preventive therapy among people living with HIV; A retrospective cohort study in Tanzania (2012-2016)

**DOI:** 10.1016/j.ijid.2020.11.192

**Published:** 2021-02

**Authors:** Werner Maokola, Bernard Ngowi, Lovetti Lawson, Masanja Robert, Michael Mahande, Jim Todd, Sia Msuya

**Affiliations:** a Ministry of Health, Community Development, Gender, Elderly and Children, Tanzania; b Institute of Public Health, Kilimanjaro Christian Medical University College Moshi Tanzania, Tanzania; c Mbeya University College of Health Sciences, Tanzania; d Zankli Medical Center, Abuja, Nigeria; e National Institute of Medical Research Mwanza, Tanzania; f London School of Hygiene and Tropical Medicine, United Kingdom

**Keywords:** ART, AntiretroviralTherapy, ARV, Antiretroviraldrugs, CTC, Careand Treatment Clinic, DTA, DataTransfer Agreement, ICF, IntensifiedTB Case Finding, INH, Isoniazid, IPT, IsoniazidPreventive Therapy, MoHCDGEC, Ministryof Health, Community Development, Gender, Elderly and Children, PLHIV, PeopleLiving with HIV, SDG, SustainableDevelopment Goals, TB, Tuberculosis, UNAIDS, UnitedNations Joint Programme on HIV/AIDS, WHO, WorldHealth Organization, Tanzania, Isoniazid preventive therapy, PLHIV and tuberculosis

## Abstract

**Background:**

Isoniazid Preventive Therapy (IPT) is a proven public health tool to reduce Tuberculosis (TB) among people living with HIV. However, its implementation in most countries is suboptimal. This retrospective study was conducted to determine the coverage and factors associated with IPT initiation to inform program scale up and improve quality of service in Tanzania and elsewhere.

**Methods:**

Retrospective cohort study design involving HIV clinics in Dar es Salaam, Iringa and Njombe regions from January 2012 to December 2016.

**Results:**

A total of 171,743 PLHIV were in the cohort from 2012 to 2016. Of these, 166,709 were involved in the analysis. Of those analyzed, 23,970 (14.38%) were ever initiated on IPT. Female sex (aOR = 1.72, 95% CI: 1.13, P < 0.001), obesity (aOR = 1.29, 95% CI:1.20−1.39, P < 0.001), WHO clinical stage II (aOR = 1.48, 95% CI: 1.42−1.55, P < 0.001), enrolment in hospitals (aOR = 1.98, 95% CI: 1.89−2.06, P < 0.001), enrolment in Njombe region (aOR = 1.25: 95% CI: 1.18−1.33, P < 0.001) and enrolment in public health facilities (aOR = 1.93: 95% CI: 1.82−2.04, P < 0.001) were associated with increased IPT uptake. Being on ART (aOR = 0.67, 95% CI: 0.65−0.70, P < 0.001) and severe nutritional status (aOR = 0.72, 95% CI: 0.60−0.88, P < 0.001) were associated with decreased IPT initiation.

**Conclusions:**

Our study documented low IPT initiation in the study area as well as documented factors which enable IPT initiation and those which impair IPT initiation. Strategies are needed to work on barriers and sustain enabling factors to improve IPT coverage.

## Highlights

• There was low IPT initiation among those who were eligible.
• Female sex, ART, obesity and WHO clinical stage II, enrolment in Njombe region and being in public hospitals were associated with increased IPT initiation.
• Strategies are needed to work on barriers and to sustain enabling factors to improve IPT initiation.

## Background

Tuberculosis (TB) among people living with HIV (PLHIV) is still a public health concern (WHO, 2018) causing increased morbidity and mortality (Dravid et al., 2019). TB disease among PLHIV threatens the benefits gained through other interventions such as antiretroviral Therapy (ART) (Diwakar et al., 2020) and such interferences may affect achieving Sustainable Development Goals and Joint United Nations Programme on HIV/AIDS goals of ending AIDS epidemics by 2030 (UNAIDS, 2014). Worldwide, in 2018, 9% of individuals diagnosed with TB disease were HIV positive. Fifty-one percent, of those with HIV had TB disease with 84% of these being on ART. There were 1.3 million, among people diagnosed with TB and 300,000 (23%) were HIV positive (WHO, 2018). Tanzania was ranked among 30 countries in the World with high TB burden in 2018. TBHIV co-infection among confirmed TB patients was 28%, and TBHIV mortality rate due to TBHIV co-infection was 29 per 100,000 population which accounted for up to 73% of all deaths among TB patients (UNAIDS, 2019, Mollel et al., 2019).

Isoniazid Preventive Therapy (IPT) which entails use of an anti-TB Drug called Isoniazid (INH) for at least 6 months, treats latent TB infection thus preventing the development of active TB disease in high risk population like PLHIV (Getahun et al., 2010). IPT is a proven public health intervention to reduce TB disease among PLHIV in real world settings. The effect is even more when IPT is used in combination with ART (Yirdaw et al., 2019, Golub et al., 2011, Shayo et al., 2017). Following this, the World Health Organization (WHO) recommends that IPT is part of comprehensive HIV care and treatment package (WHO, 1998).

Despite available evidence on the benefits of IPT on PLHIV, use of this effective preventive tool is suboptimal (Lester et al., 2010, Grant et al., 2005, Ayele et al., 2015, Makoni et al., 2015). Suboptimal implementation of IPT is equally present even in parts of the world hard-hit by both HIV and TB such as Sub-Saharan Africa (Wilkinson and Davies, 1997) where service is needed most. In 2017, IPT among PLHIV Worldwide ranged from 1% in Eswatini to 53% in South Africa (WHO, 2018). Reasons for low IPT coverage range from health provider factors, to client factors, to general health system factors (Lester et al., 2010, Mindachew et al., 2014).

Tanzania has been implementing the use of IPT among PLHIV in HIV care and treatment clinics to prevent development of TB diseases since 2011 (Sabasaba et al., 2019). Here we describe characteristics of PLHIV who were screened TB negative (prerequisite for IPT initiation), determine characteristics of PLHIV who initiated IPT as well as determine factors associated with IPT initiation as IPT has been recently integrated in HIV care and treatment clinics in Tanzania. The findings from this study will inform IPT program scale up in Tanzania and elsewhere.

## Methods

### Study design and setting

We described a secondary data analysis of a retrospective cohort study using the STROBE checklist (Cuschieri, 2019). Anonymized data from all PLHIV enrolled in 315 HIV care and treatment clinics (CTCs) in Dar es Salaam, Iringa and Njombe regions over the period from January 2012 to December 2016. Iringa and Njombe regions have higher HIV prevalence, above the national average (URT, 2018) and Dar es Salaam to has the highest TB case notifications in the country (Said et al., 2017).

### Study population

This study used retrospective data collected from PLHIV enrolled in HIV care and treatment clinics in the three regions enrolled in the specified study period. PLHIV who had TB disease diagnosis before CTC enrolment and those who attended clinics in health facilities which never implemented IPT throughout the study period were excluded from the analysis.

### Data collection

In Tanzania TB management is integrated in HIV care and treatment services to reduce TB among PLHIV. Information regarding the cohort is detailed elsewhere (Maokola et al., 2020). In summary, three interventions to reduce TB are implemented in HIV care and treatment clinics; these are Intensified TB case finding (ICF), IPT and TB infection prevention and control. During every clinic visit PLHIV are screened for active TB using symptoms/signs as part of ICF. Those who are found with at least one symptom or sign of TB undergo further laboratory and radiological investigations to diagnose TB disease. Those who are found to have no symptoms/signs of active TB are offered IPT for at least 6 months provided other criteria are fulfilled. TB infection and prevention measures are also implemented in all HIV care and treatment clinics to reduce TB infection transmission.

CTCs have patient-level data collection containing data on every visit made by PLHIV. For up to 80% of clinics, data including those related to TB are electronically captured at health facility level into an electronic database called the CTC2 database. Data from the CTC2 database is transferred to the national level database (CTC3) in real time, where demographic and clinical information of each PLHIV identified only by their unique identification CTC number is available for analysis.

Information for PLHIV enrolled in the three study regions were extracted from the CTC3 database. The extracted database had the following variables: The extracted database contained study participant unique identification, information regarding demographic data of the study participants, characteristics of the health facilities, clinical information of the study participants including ART information and TB management. The data were cleaned by checking missing values and abnormal entries. STATA version 14 was used for data analysis. Demographic and clinical variables were used as independent variables and IPT status (received/not received) was the dependent variable. PLHIV with missing data for covariates were excluded from the analyses on a case by case basis, giving a complete case analysis. Missing data were most frequent for clinical characteristics which are not routinely measured in CTC, although these are available at baseline (enrolment into the clinic). As less than 1% of PLHIV were missing these clinical characteristics, the analysis was conducted based on complete case analysis.

Descriptive analysis was used to describe baseline characteristics of study participants and uptake of IPT. Multivariate logistic regression was used to determine factors associated with IPT initiation status involving only exposure variables which were statistically significant during univariate analysis. Multilevel analysis was done to account for cluster effects at the health facility. A statistical significance level of 5% was used.

## Results

### Baseline characteristics of study participants

**Figure 1:**
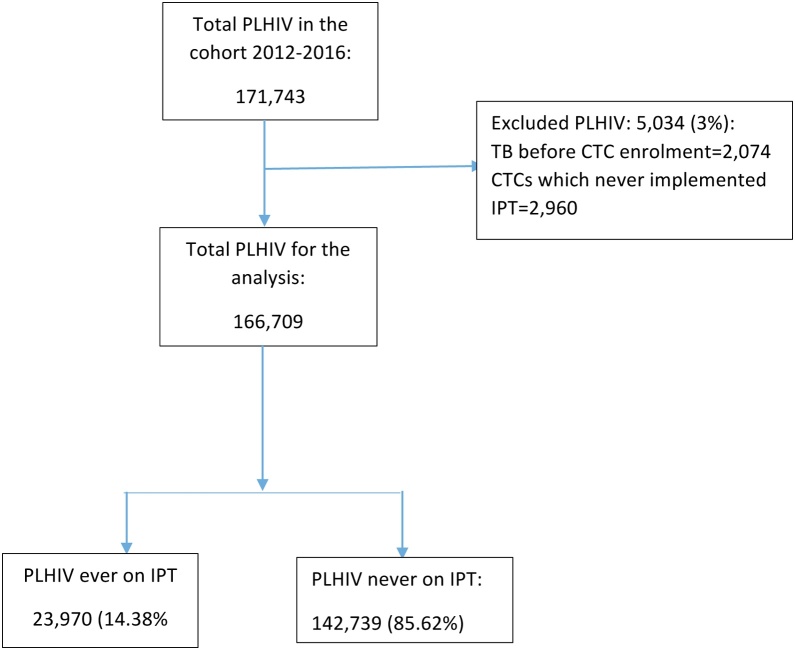
IPT initiation cascade among PLHIV attending CTC during the follow up period.

A total 171,743 PLHIV were enrolled in CTCs in the 3 regions during the study period from 2012 to 2016. Four (UNAIDS, 2014) percent of study participants were excluded from the analysis as they either had TB before CTC enrolment or they were in clinics which never implemented IPT throughout the study period. Hence, 166,709 were included in the final analysis; of these 23,970 were initiated on IPT (Figure 1).

Most of the study participants were females (68.57%), aged 25−49 (71.98%), with walking functional status (95.95%), not on ART (64.79%), with normal nutritional status (92.01%) and normal BMI (56.33%). Study participants were also more likely to be WHO clinical stage I (36.58%), enrolled in 2014 (21.20%), from dispensary level (37.71%), with good adherence to ARV (98.54%), enrolled in Dar es Salaam (72.12%) and from public health facilities (72.82%) (Table 1).

**Table 1:** Baseline characteristics of study participants (N = 166,709).

Characteristics	Frequency, n	Percentage, %
**IPT status**		
Never on IPT	142,739	85.62%
Ever on IPT	23,970	14.38%
**Sex**		
Male	52,391	31.43%
Female	114,318	68.57%

**Age (Years):** Mean = 34 SD = 13 Range = 0−95		
0−9	7520	4.51%
10−19	7342	4.40%
20−24	15,884	9.53%
25−49	119,993	71.98%
+50	15,961	9.57%

**Functional status**		
Ambulatory	5360	3.23%
Bedridden	1.366	0.82%
Walking	159,246	95.95%

**ART status**		
ART-No	107,219	64.79%
ART-Yes	58,274	35.21%

**Nutritional status**		
Normal	146,320	92.01%
Moderate	10,945	6.88%
Severe	1755	1.10%

**BMI**		
Underweight	19,006	17.53%
Normal	61,081	56.33%
Overweight	19,316	17.81%
Obesity	9036	8.33%

**WHO clinical stage**		
Stage I	60,191	36.58%
Stage II	39,252	23.85%
Stage III	52,667	32.00%
Stage IV	12,455	7.57%

**Enrolment year**		
2012	30,096	18.05%
2013	32,975	19.78%
2014	35,344	21.20%
2015	32,047	19.22%
2016	36,247	21.47%

**Health Facility level**		
Dispensary	62,865	37.71%
Health Center	44,561	26.73%
Hospital	59,283	35.56%

**ARV adherence**		
Good	31,201	98.54%
Poor	463	1.46%

**Region**		
Dar es Salaam	120,383	72.12%
Iringa	18,162	10.89%
Njombe	28,164	16.89%

**Health Facility ownership**		
Private	45,309	27.18%
Public	121,400	72.82%

### Characteristics of study participants initiated on IPT

Most of those initiated on IPT were females (71.41%), aged 25−49 years (77.40%), with walking functional status (97.25%), not on ART (72.23%), with normal nutritional status (93.31%) and attended in hospitals (48.86%). Moreover, most of those initiated on IPT were of normal BMI (57.59%), with WHO clinical stage III (34.39%), enrolled in 2014 (22.80%), with good adherence to ARVs (98.98%) and enrolled in public health facilities (84.36) (Table 2).

**Table 2:** Characteristics of study participants initiated on Isoniazid Preventive Therapy.

Covariate	IPT status		Total
	IPT-No	IPT-Yes	
**Sex**			
Male	45,539(31.90%)	6852(28.59%)	52,391
Female	97,200(68.10%)	17,118(71.41%)	114,318

**Age (Years):**			
0−9	6987(4.90%)	533(2.22%)	7520
10−19	6637(4.65%)	705(2.94%)	7342
20−24	14,164(9.92%	1720(7.18%)	15,884
25−49	101,441(71.07%)	18,552(77.40%)	119,993
+50	13,501(9.46%)	2460(10.26%)	15,961

**Functional status**			
Ambulatory	4844(3.41%	516(2.16%)	5360
Bedridden	1226(0.86%)	140(0.59%)	1366
Walking	136,039(95.73%	23,207(97.25%)	159,246

**ART status**			
ART-No	90,009(63.52%)	17,210(72.23%)	107,219
ART-Yes	51,690(36.48%)	6584(27.67%)	58,274

**Nutritional status**			
Normal	124,761(91.79%)	21,559(93.31%)	146,320
Moderate	9558(7.03%)	1387(6.00%)	10,945
Severe	1596(1.17%)	159(0.69%)	1755

**BMI**			
Underweight	16,376(18.05%)	2630(14,85%)	19,006
Normal	50,879(56.08%	102,020(57.59%)	61,081
Overweight	15,989(17.62%)	3327(18.78%)	19,316
Obesity	7479(8.24%)	1557(8.79%)	9036

**WHO clinical stage**			
Stage I	52,411(37.20%)	7780(32.87%)	60,191
Stage II	32,948(23.39%)	6304(26.63%)	39,252
Stage III	44,526(31.60%)	8141(34.39%)	52,667
Stage IV	11,008(7.81%)	1447(6.11%)	12,455
Enrolment Year			
2012	25,781(18.06%)	4315(18.00%)	30,096
2013	28,045(19.65%)	4930(20.57%)	32,975
2014	29,880(20.93%)	5464(22.80%)	35,344
2015	27,297(19.12%)	4.750(19.82%)	32,047
2016	31,736(22.23%)	4511(18.82%)	36,247

**Health Facility level**			
Dispensary	56,931(39.88%)	5934(24.76%)	62,865
Health Center	38,236(26.79%)	6325(26.39%)	44,561
Hospital	47,572(33.33%)	11,711(48.86%)	59,283

**ARV adherence**			
Good	27,611(98.48%)	5590(98.98%)	31,201
Poor	426(1.52%)	37(1.02%)	463

**Region**			
Dar es Salaam	102,233(71.62%)	18,150(75.12%)	120,383
Iringa	17,176(12.03%)	986(4.11%)	18,162
Njombe	23,330(16.34%)	4834(20.17%	28,164

**Health Facility ownership**			
Private	41,559(29.12%	3750(15.64%)	45,309
Public	101,180(70.88%)	20,220(84.36%)	121,400

### Determinants of IPT initiation

In multivariate analysis; sex, functional status, ART status, nutritional status, BMI, WHO Clinical stage, health facility type, adherence to ARVs, region and health facility ownership, had statistical significant association with IPT initiation. Female sex (aOR = 1.72, 95% CI: 1.13, P < 0.001), obesity (aOR = 1.29, 95% CI:1.20−1.39, P < 0.001), WHO clinical stage II (aOR = 1.48, 95% CI: 1.42−1.55, P < 0.001), enrolment in hospitals (aOR = 1.98, 95% CI: 1.89−2.06, P < 0.001), enrolment in Njombe region (aOR = 1.25: 95% CI: 1.18−1.33, P < 0.001) and enrolment in public health facilities (aOR = 1.93: 95% CI: 1.82−2.04, P < 0.001) were associated with increased IPT uptake. Being on ART (aOR = 0.67, 95% CI: 0.65−0.70, P < 0.001) and severe nutritional status (aOR = 0.72, 95% CI: 0.60−0.88, P < 0.001) were associated with decreased IPT initiation. (Table 3).

**Table 3:** Factors associated with IPT initiation (N = 166,709).

Covariate	Univariate analysis	Multivariate analysis
	cOR, 95% CI, P-value	aOR, 95% CI, P-value
**Sex**		
Male	1	1
Female	1.20(1.16−1.23), P < 0.001	1.72(1.13−1.22), P < 0.001

**ARV status**		
ART-No	1	1
ART-Yes	40.13(33.05−48.73), P < 0.001	0.67(0.65−0,70), P < 0.001

**Nutritional status**		
Normal	1	1
Moderate	0.82(0.77−0.87)	0.80(0.74−0.86)
Severe	0.53(0.45−0.62), P < 0.001	0.72(0.60−0.88), P < 0.001

**BMI**		
Underweight	1	1
Normal	1.31(1.25−1.37)	1.25(1.18−1.31)
Overweight	1.38(131−1.46)	1.27(1.20−1.36)
Obesity	1.36(1.27−1.46), P < 0.001	1.29(1.20−1.39), P < 0.001

**WHO clinical stage**		
Stage I	1	1
Stage II	1.23(1.18−1.27)	1.48(1.42−1.55)
Stage III	1.13(1.10−1.17)	1.23(1.18−1.28)
Stage IV	0.79(0.75−0.84), P < 0.001	0.88(0.81−0.95), P < 0.001

**Health Facility level**		
Dispensary	1	1
Health Center	1.43(1.38−1.49)	1.73(1.66−1.82)
Hospital	2.04(1.97−2.11), P < 0.001	1.98(1.89−2.06), P < 0.001

**Region**		
Dar es Salaam	1	1
Iringa	0.32(0.30−0.35)	0.39(0.35−0.42)
Njombe	1.17(1.13−1.21), P < 0.001	1.25(1.18−1.33), P < 0.001

**Health Facility ownership**		
Private	1	1
Public	2.21(2.13−2.30), P < 0.001	1.93(1.82−2.04), P < 0.001

*95% Confidence Interval.

## Discussion

The study registered low uptake of IPT among PLHIV. Female sex, ART, obesity, WHO clinical stage, enrolment in Njombe region, and being in public hospitals were the determinants of IPT initiation.

Only about 80% of the HIV clinics in the country had an electronic HIV database and hence were capable of contributing data to the national database. Health facilities at low levels did not have electronic databases and were unable to contribute data. Secondly, the analysis included 3 regions out of 26 regions. These regions were purposively chosen as they had the highest HIV and TB prevalence. They may therefore not represent a true picture of other regions which were not involved in the analysis.

Our study registered lower IPT initiation compared to other studies which were also conducted in routine settings in Zimbabwe, Ethiopia and Nepal (Makoni et al., 2015, Nyathi et al., 2019, Yirdaw et al., 2019, Teklay et al., 2016, Wesen and Mitike, 2012, Dhungana et al., 2019). These studies documented IPT initiation ranging from 20% to 54%. The observed low IPT initiation in the current study needs further research. Nevertheless, most of the studies, despite being conducted in routine settings like our study, involved small numbers of health facilities. None of the above studies involved more than 20 high level health facilities. Our study involved a total of 315 in the 3 regions containing a mix of high and low level health facilities. Shortage of INH, fear of INH resistance, lack of confidence in ruling out TB disease, lack of commitment among providers and hesitancy in accepting IPT among clients were among the barriers for IPT implementation documented in several other studies (Nyathi et al., 2019, Teklay et al., 2016, Wesen Denegetu and Lovely Dolamo, 2014). Improvement in recording and reporting in order to identify PLHIV who are eligible for IPT, health education to curb fear of adherence and side effects among PLHIV, interventions to reduce staff workload and avoiding INH stockout are the interventions recommended to improve IPT coverage (Teklay et al., 2016, Ogunsola et al., 2019, Legese et al., 2020) which could be applied in our setting as well.

Consistent with our study, other studies also documented a high IPT initiation among females, ART recipients, PLHIV enrolled in public hospitals and PLHIV with relatively good health (WHO clinical stage II) (Nyathi et al., 2019, Trinh et al., 2016, Van Ginderdeuren et al., 2019, Ayele et al., 2017). High uptake among females could be due to more health information for women during medical encounters such as maternal and child health services (Wesen Denegetu and Lovely Dolamo, 2014). ART experience and recipients’ good health may increase confidence for IPT initiation among health care providers.

In our study determinants of IPT initiation were female sex, ART, obesity and WHO clinical stage II. Other IPT initiation determinants were enrolment in the Njombe region and in public hospitals. As stated above, females have a higher threshold embracing health services due to their health-seeking behavior (Thompson et al., 2016) as well as their increased opportunity to encounter health care providers for reproductive health (Wesen Denegetu and Lovely Dolamo, 2014). Other studies also found an association between female sex and IPT uptake (Adeniyi et al., 2019). We also found IPT initiation to be associated with health individuals (WHO clinical stage II). Our finding is in agreement with what was found in Kenya (Nyathi et al., 2019). The reason for avoiding IPT in severely ill PLHIV can be attributed to fear of clinicians due to difficulty in ruling out TB disease in this population as speculated in the Kenyan study (Nyathi et al., 2019). High IPT initiation in hospitals and public health facilities could be explained by the fact that IPT was rolled out gradually starting with Hospitals. Higher initiation in public versus in private health facilities could be due to a slower pace of private health facilities in implementing new policies in comparison with public owned health facilities (Buregyeya et al., 2017). Higher IPT initiation in Iringa and Dar es Salaam in comparison with Njombe explains dynamics in IPT implementation, which also differs from health facility to health facility, as reported in another study showing different implementation levels in different places (Dhungana et al., 2019, Omondi et al., 2018, Ngugi et al., 2020).

## Conclusion

Our study, which used routine HIV data from 315 clinics, documented low IPT initiation proportion among those who were enrolled in HIV care and eligible for IPT in Dar es Salaam, Iringa and Njombe during the study period. Variations in IPT initiation among regions signal different dynamics affecting IPT uptake in different regions and hence require customized approaches in quality improvement. Implementation research is needed to understand health systems as well as cultural barriers in the uptake of IPT intervention and design quality improvement initiative accordingly.

## Declarations

This article is an original research which is one of the required number of published articles for PhD training of the first author. We declare that this article has not been submitted to any other journal for publication.

## Ethics approval and consent to participate

The study used routine HIV data from Tanzania. Hence, the study did not have contact with human subjects. Approval for conducting the study was obtained from Kilimanjaro Christian Medical University College in Moshi Tanzania. Permission to use routine HIV data was obtained from Ministry of Health, Community Development, Gender, Elderly and Children (MoHCDGEC) Tanzania by signing Data Transfer Agreement (DTA).

## Consent for publication

Authors of the study collaboratively give permission to the publisher to publish the study findings as presented by the authors.

## Availability of data and materials

Data and other materials used for the study belong to the MoHCDGEC Tanzania. Permission to access data and other materials used for the study can be obtained from the Permanent Secretary MoHCDGE in Tanzania.

## Competing interests

Authors of the study declare that there is no conflict of interest involved in conducting the study.

## Funding

The study was supported by the SEARCH(Sustainable Evaluation through Analysis of Routinely Collected HIV data) Project. We acknowledge funding by the Bill & Melinda Gates Foundation grant number OPP1084472 entitled“Using routinely collected public facility data for program improvement in Tanzania, Malawi and Zambia.” This paper has been produced as part of the PhD studies of WM for the evaluation of TB services in HIV care and treatment services in Tanzania was financed collaboratively by the Government of Tanzania and US President’s Emergency Plan for AIDS Relief (PEPFAR).

## Authors’ contributions

WM conceived the study idea, developed first draft of the study protocol, developed data analysis plan and developed the first draft of the manuscript. BN, LL, JT, MM and SM reviewed study protocol, data analysis plan and study manuscript. WM, BN, LL, JT, MM and SM conducted data management. All authors agreed on the final version of the manuscript.
